# Structural gender inequities in dental specialty training in Türkiye: evidence from a national cross-sectional study

**DOI:** 10.1186/s12909-026-09032-x

**Published:** 2026-03-17

**Authors:** Esra Balkanlıoğlu, Aykut Can Balkanlıoğlu, Aliye Kamalak

**Affiliations:** 1 Department of Endodontics, Kahramanmaras Sutcu Imam University, Avsar Campus West Ring Road Boulevard No: 251/A Onikisubat, Kahramanmaras, Turkey; 2 Department of Oral and Maxillofacial Surgery, Kahramanmaras Sutcu Imam University, Kahramanmaras, Turkey

**Keywords:** Dental specialty education, Gender-based inequities, Dental workforce, Artificial intelligence, Academic dentistry

## Abstract

**Background:**

This study investigated gender-based disparities in specialty preferences among dental students in Türkiye and evaluated the predictive performance of an artificial intelligence–based gender classification tool (NamSor). This study aimed to determine how these disparities shape specialty distribution and to assess whether AI-based gender identification aligns with verified data.

**Methods:**

A cross-sectional descriptive design was employed using publicly available data from Turkish university websites and the Council of Higher Education. Gender was manually verified for all specialty students and compared with NamSor’s predictions. Statistical analyses included Chi-square tests, Bonferroni adjustments, Kappa coefficient, accuracy metrics, and ROC curve analysis to determine the agreement between actual and predicted gender classifications.

**Results:**

Significant gender disparities were observed across specialties (χ²=443.55; *p* < 0.001). Female students were predominantly represented in Pediatric Dentistry (17.9%) and Restorative Dentistry (12.4%), whereas males were concentrated in Oral and Maxillofacial Surgery (26.5%). Gender distribution also differed according to university type. NamSor demonstrated moderate accuracy (67.9%), with higher sensitivity in females (75.1%) than in males (53.6%). The Kappa coefficient was 0.283, indicating a fair agreement between the actual and predicted gender. Misclassification was notable, as 24.9% of women and 46.4% of men were incorrectly classified. The AUC was 0.64, suggesting a modest discrimination ability.

**Conclusions:**

Gender-based imbalances persist in dental specialty education in Türkiye, particularly in surgical fields, where women remain underrepresented. The modest performance of the NamSor algorithm underscores that AI-based name classification should be regarded as an exploratory method and applied only with caution, while verified administrative data remain the reference standard for gender identification. These findings highlight the importance of equity-focused policies, mentorship initiatives, and gender-sensitive strategies to support balanced participation and career development in dental education.

**Graphical Abstract:**

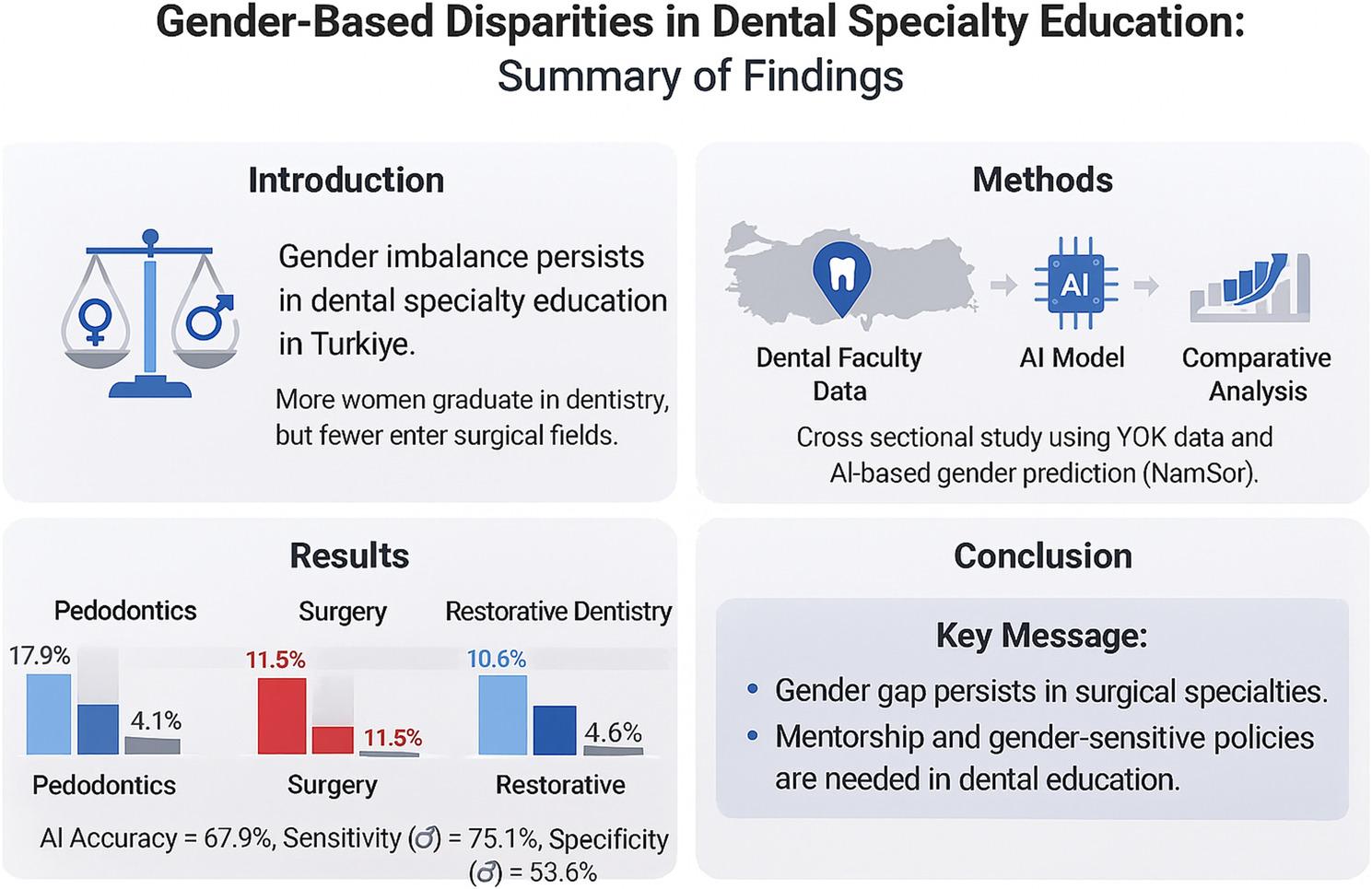

## Background

Gender equality is regarded as a fundamental condition for quality, equity, and sustainability in health systems. Ensuring that female and male have equal opportunities in accessing healthcare services plays a critical role not only in terms of individual rights, but also in enhancing societal welfare. However, the literature shows that the concept of gender equality is defined and measured differently. The measurement methods, indicators used, and distinctions between “difference” and “inequality” directly affect the evaluations made [1].

The number of women in dental education has increased worldwide. However, this increase has not been reflected equally in specialist fields or academic career ranks. Women are concentrated in lower-level positions, and their representation decreases as seniority increases [2]. In recent years, although the number of graduates has increased, representation has decreased at advanced academic stages [3]. Gender differences in career choices and expectations become apparent at an early age. While female students tend to focus on work-life balance, secure employment, and interpersonal relationships, male students prioritize technical skills, entrepreneurship, and high career advancement prospects [4]. Self-efficacy beliefs and outcome expectations play critical roles in career choice, and these psychosocial components also influence specialty selection [5].

The representation of women significantly decreases in surgical specialties with high intensity. Oral, dental, and maxillofacial surgeries are typical examples of this situation. The number of females remains limited, and their academic-career durations are shorter. Although indicators such as academic rank and h-index appear higher in men, when years of seniority are considered, gender ceases to be an independent factor [6]. Global-scale analyses show that these imbalances stem not only from personal preferences but also from structural factors such as geographical conditions, mentoring opportunities, and work-life balance [7].

Perceptions of instructor gender in educational settings can also shape students’ career orientation. Students view female instructors as more inclusive, understanding, and supportive; however, they do not perceive any gender-based differences in knowledge level or technical competence [8]. This finding suggests that role models are more effective in terms of perceived social support than peer models. A similar inequality is observed in academic productivity. It has been determined that, among the most-cited dentistry articles of recent decades, female researchers are particularly underrepresented as “senior authors.” Although the rate of representation has increased over time, an imbalance persists [9]. Low visibility in publications is a major barrier to career advancement and the transition to academic leadership for female researchers in India. In summary, despite the strong representation of women at the undergraduate level in dentistry, inequalities persist in specialty training, surgical branches, and academic career advancement. The causes of these disparities extend beyond individual choices and include structural factors such as mentorship, working conditions, and the academic climate.

In the context of dental education, understanding gender-based disparities is not merely a matter of representation but also a determinant of how educational systems allocate opportunities, mentorship, and resources. The unequal participation of women in surgical specialties and academic stages may lead to a lack of diverse perspectives on curriculum design, clinical decision-making, and faculty development [10]. International frameworks, including the American Dental Education Association Commission on Change and Innovation in Dental Education [11] and the World Health Organization Global Oral Health Strategy (2022) [12], emphasize the integration of gender-sensitive approaches into dental education curricula. Therefore, examining these disparities within the Turkish context provides valuable insights into aligning national dental education policies with global equity standards [12].

This study examined gender-based disparities in dental specialty education in Türkiye and assessed how well an artificial intelligence–based name classification model (NamSor) aligns with verified gender data. By combining empirical educational data with AI analysis, this study provides novel insights into structural inequities in specialty training and their implications for dental education policies.

## Methodology

**Fig. 1:**
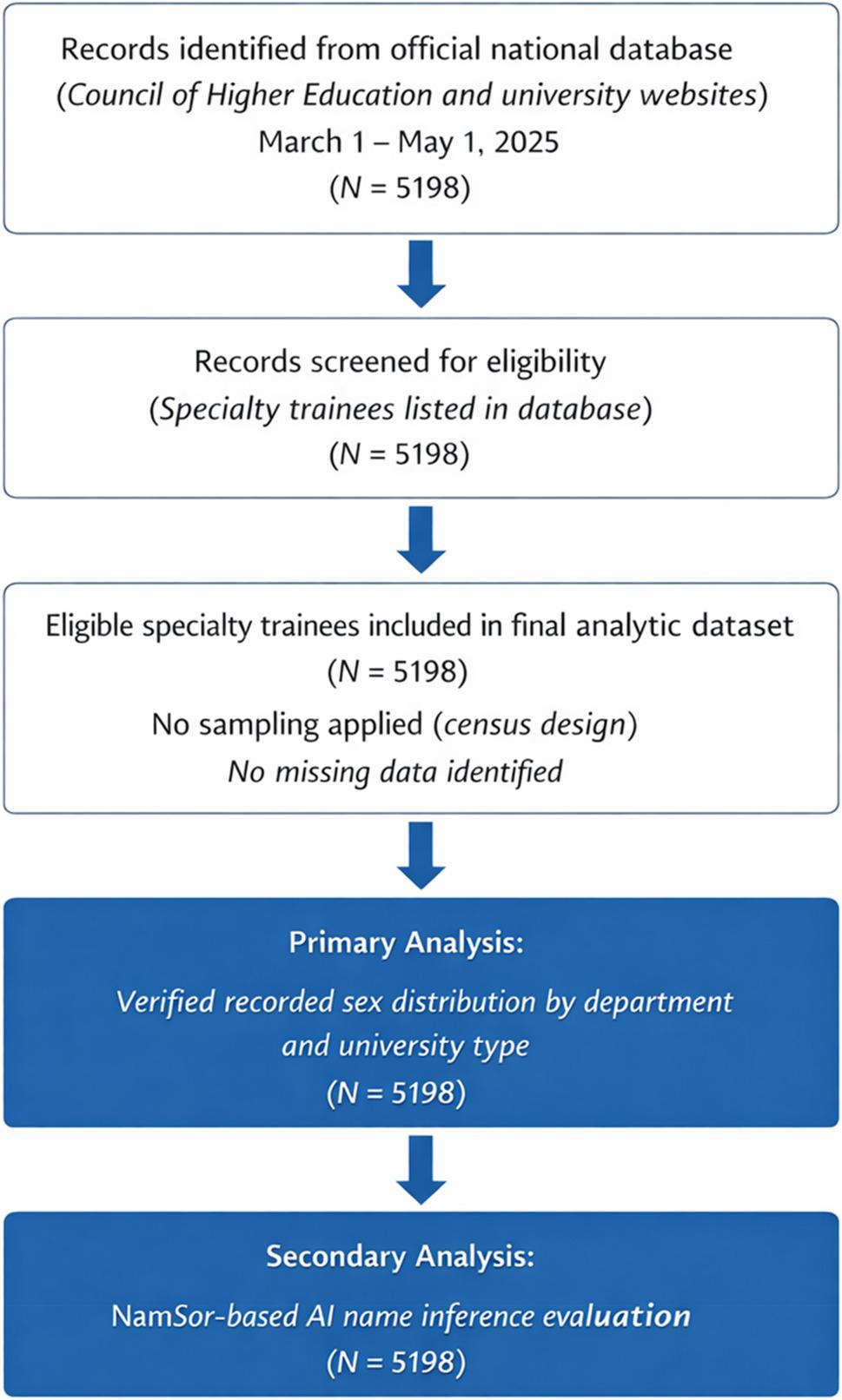
STROBE flow diagram of participant identification and inclusion. *All specialty trainees listed in the official national database during the study period were included (*N* = 5198). The dataset represents a census design with no missing values. Both verified recorded sex analyses and NamSor-based AI evaluations were conducted using the same population.

This cross-sectional descriptive study examined the gender distribution of students receiving specialist education in Turkish dental faculties in Türkiye. As all information was publicly available, ethics committee approval was not required for this study. This cross-sectional study was designed and reported in accordance with the Strengthening the Reporting of Observational Studies in Epidemiology (STROBE) guidelines for observational research (Fig. 1).

### Data sources and collection method

Publicly available data were used to determine the departments of dentistry specialty students at all state and foundation universities. In Türkiye, “Foundation Universities” refer to privately established, non-profit higher-education institutions operating under public higher-education regulations. The names of the students in each specialty program, their fields of specialty, gender, and affiliated university were recorded. The category “General practitioner” reflects individuals listed in the administrative database without formal enrollment in a recognized specialty training program and was retained to ensure the completeness of the official dataset. The data registered on the Council of Higher Education (YÖK) website were used for collection, and the records created for each university were examined individually (https://yokatlas.yok.gov.tr/lisans-bolum.php?b=10049). Data were recorded between March 1 and May 1, 2025. This study aimed to include the entire population of specialty trainees listed in the official national database during the data collection period. Therefore, no sampling procedure was applied, and the dataset represented a census of eligible individuals. No missing data were identified in the officially recorded variables used in the primary analysis.

### Gender identification process

By examining the data of dentistry specialty students registered with the Council of Higher Education, the sex classification used in this study was manually verified and found to be fully consistent with officially recorded administrative data. In addition, we used the NamSor gender identification tool to assess how well the results matched the definitive results and to evaluate the usefulness of AI-based gender identification tools. NamSor is a name-based gender inference algorithm that estimates gender probabilities using large-scale global datasets that link first and last names to demographic patterns. This model relies on linguistic, cultural, and geographic name distributions to assign probabilistic gender classifications. Therefore, its performance may vary depending on the naming conventions and regional heterogeneity. Gender was operationalized in a binary framework (female/male) based on officially recorded data. The dataset did not include information on non-binary or gender-diverse identity.

In this study, the variables available in the institutional records corresponded to legally registered sex (female/male). For consistency with the equity-focused literature, the term “gender” is used throughout the manuscript when discussing structural disparities, while acknowledging that the empirical classification reflects recorded sex.

The inclusion of NamSor was not intended to replace or validate the manually verified gender data, but rather to critically assess the reliability of automated gender inference tools in contexts where verified gender information may not be available. Therefore, NamSor analyses were conducted as a methodological robustness and tool performance evaluation rather than as a basis for the primary inferential findings of the study. We compared the accuracy of the gender identification tools with the definitive results obtained from the Council of Higher Education.

### Data analysis

Data analysis was performed using the Statistical Package for the Social Sciences (SPSS) 26.0 Statistics software. To evaluate the relationship between dental departments and actual gender, the Pearson Chi-Square test was applied; the same method was also used to examine the relationship between university type and actual gender. Subsequently, the relationships between dental departments, university types, and gender distributions predicted by the NamSor software were analyzed using the Pearson Chi-Square test. To determine differences between groups, the Bonferroni test was used, and different letters presented in the tables and figures indicate statistical differences between groups. The Kappa coefficient was used to assess the agreement between the actual and NamSor-predicted gender. In addition, accuracy, sensitivity, specificity, and precision metrics were calculated to demonstrate the classification performance. Furthermore, to evaluate the overall classification power of the NamSor predictions, the ROC curve and area under the curve (AUC) values were examined. The interpretation of the Kappa coefficient was carried out according to the classification developed by Landis and Koch (1977); accordingly, a Kappa value below 0 indicates poor agreement, 0.00–0.20 slight agreement, 0.21–0.40 fair agreement, 0.41–0.60 moderate agreement, 0.61–0.80 substantial agreement, and 0.81–1.00 almost perfect agreement [13]. In all statistical analyses, significance levels were set at *p* < 0.05 and *p* < 0.01.

## Results

### Comparison of actual and predicted (NamSor) gender distributions by dentistry departments and university types

**Fig. 2:**
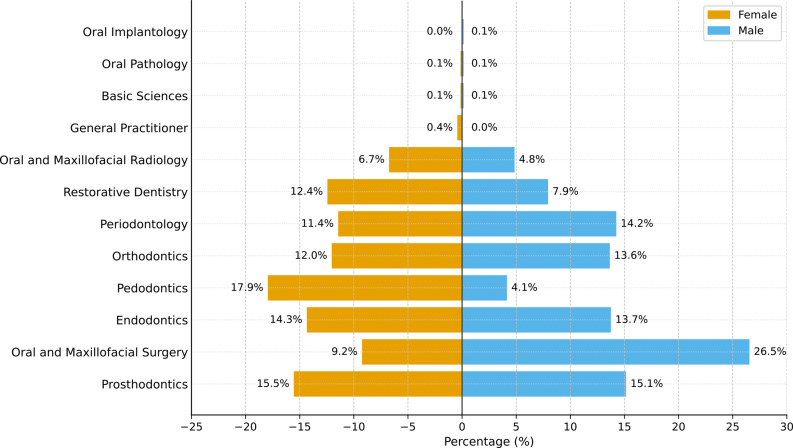
Actual gender distribution by departments of dentistry (Total sample size: *N* = 5198). * The percentages represent the proportions within each gender group. Values for women (*n*, %) are shown on the left and those for men (*n*, %) are shown on the right. The values above the bars represent the percentage rates

A comparison of the actual gender distribution by dentistry department and university type is presented in Table 1. Graphs showing the gender distribution by department and university type are shown in Fig. 2.

**Table 1:** Comparison of actual gender distribution by dentistry departments and types of public institutions

Variable		Gender				Test statistic
		Female		Male		
		Number	%	Number	%	
Departments	Prosthodontics	538^a^	15.5	259^a^	15.1	χ²=443.55
	Oral and Maxillofacial Surgery	320^a^	9.2	456^b^	26.5	*p* < 0.001**
	Endodontics	497^a^	14.3	235^a^	13.7	
	Pediatric Dentistry	624^a^	17.9	70^b^	4.1	
	Orthodontics	419^a^	12.0	233^a^	13.6	
	Periodontology	397^a^	11.4	244^b^	14.2	
	Restorative Dentistry	430^a^	12.4	135^b^	7.9	
	Oral and Maxillofacial Radiology	233^a^	6.7	83^b^	4.8	
	General practitioner	13^a^	0.4	0^b^	0.0	
	Department of Basic Sciences	5^a^	0.1	2^b^	0.1	
	Oral Pathology	3^a^	0.1	1^b^	0.1	
	Oral Implantology	0^a^	0.0	1^b^	0.1	
Type of university	State university	3330^a^	95.7	1668^b^	97.0	χ²=5.036
	Foundation university	149^a^	4.3	51^b^	3.0	*p* = 0.025*

Different superscript letters (^a, b^) within the same row indicate statistically significant differences between groups based on Bonferroni-adjusted comparison tests

*p < 0.05; **p < 0.01; χ²=Chi-Square Test

A statistically significant relationship was found between the dentistry departments and recorded sex (χ²=443.55; *p* < 0.001). The findings indicate that the gender distribution within the departments is not equal. The department with the highest proportion of females was Pedodontics, where the proportion of females was 17.9% (*n* = 624), while the proportion of males was only 4.1% (*n* = 70). Similarly, in the Restorative Dental Treatment (12.4% female, 7.9% male) and Oral, Dental, and Maxillofacial Radiology (6.7% female, 4.8% male) departments, the proportion of females was higher than that of males. Conversely, in the Oral, Dental, and Maxillofacial Surgery department, the proportion of males was noticeably higher at 26.5% (*n* = 456), compared to only 9.2% (*n* = 320) for females. Periodontology (14.2% male, 11.4% female) and Oral Implantology (0.1% male) are also fields where the male concentration is relatively higher.

When examined by university type, the proportion of females at state universities was 95.7% (*n* = 3330), and the proportion of males was 97.0% (*n* = 1668); at foundation universities, the proportion of females was 4.3% (*n* = 149), and the proportion of males was 3.0% (*n* = 51). A significant relationship was also found between university type and gender (χ²=5.036; *p* < 0.05). These findings reveal that there are clear differences in the department preferences of female and male dentists and that there are also significant changes in gender distribution based on university type. These findings indicate that gender-based specialty concentration may shape the future distribution of clinical expertise and academic representation in Turkish dental faculties. Differences between state and foundation universities should be interpreted cautiously, as structural variations in institutional size, funding models, and regional distribution may influence specialty-enrollment patterns.

**Fig. 3:**
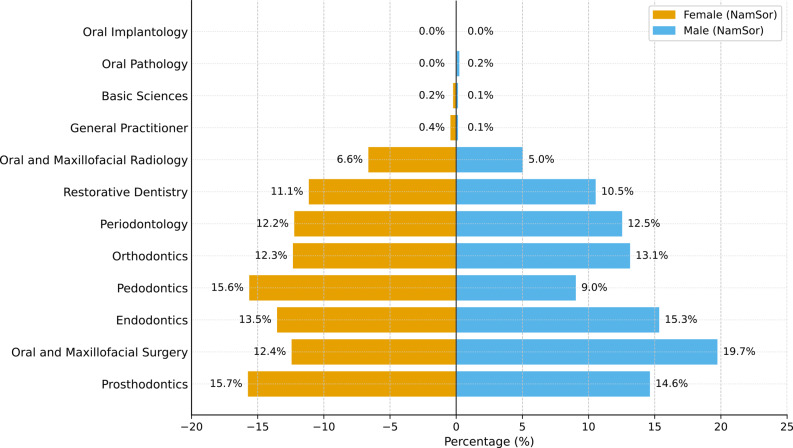
Gender (NamSor) distribution by dentistry departments (Total sample size: *N* = 5198). Percentages represent proportions within each gender group

Table 2 compares the estimated gender distribution according to NamSor with dentistry departments and university types. The distribution by department is illustrated in Fig. 3.

**Table 2:** Comparison of gender (NamSor) distribution by dentistry departments and types of public institutions

Variable		Gender (NamSor)				Test statistic
		Female		Male		
		Number	%	Number	%	
Departments	Prosthodontics	535^a^	15.7	262^a^	14.6	χ²=99.260
	Oral and Maxillofacial Surgery	423^a^	12.4	353^b^	19.7	*p* < 0.001**
	Endodontics	459^a^	13.5	273^a^	15.3	
	Pediatric Dentistry	533^a^	15.6	161^b^	9.0	
	Orthodontics	418^a^	12.3	234^a^	13.1	
	Periodontology	417^a^	12.2	224^a^	12.5	
	Restorative Dentistry	378^a^	11.1	187^a^	10.5	
	Oral and Maxillofacial Radiology	226^a^	6.6	90^b^	5.0	
	General practitioner	12^a^	0.4	1^b^	0.1	
	Department of Basic Sciences	6^a^	0.2	1^a^	0.1	
	Oral Pathology	1^a^	0.0	3^a^	0.2	
	Oral Implantology	1^a^	0.0	0^a^	0.0	
Type of university	State university	3264^a^	95.7	1734^b^	96.9	χ²=4.096
	Foundation university	145^a^	4.3	55^b^	3.1	*p* = 0.43*

The letters in the table indicate statistical differences between the groups

*p < 0.05; **p < 0.01; χ²=Chi-Square Test

The analysis revealed a statistically significant relationship between the departments and estimated gender (χ²=99.260; *p* < 0.05). In the Pedodontics department, the proportion of females was significantly higher at 15.6% (*n* = 533) compared to males at 9.0% (*n* = 161). Similarly, in the Prosthetic Dental Treatment (15.7% female, 14.6% male) and endodontics (13.5% female, 15.3% male) departments, the proportion of females was relatively higher. In contrast, in the Oral, Dental, and Maxillofacial Surgery department, the proportion of males was higher at 19.7% (*n* = 353) compared to 12.4% of females (*n* = 423). In the Orthodontics, Periodontology, and Restorative Dental Treatment departments, the distribution between females and males was similar.

From the perspective of university type, 95.7% of females (*n* = 3264) and 96.9% of males (*n* = 1734) are at state universities, while at foundation universities, 4.3% of females (*n* = 145) and 3.1% of males (*n* = 55) are represented. A statistically significant relationship was also found between university type and the NamSor estimated gender (χ²=4.096; *p* < 0.05). These findings indicate that there are notable differences in gender distribution by department and that the proportions of female and male specialty students at state and foundation universities differ significantly.

### Comparison and concordance analysis of actual gender and NamSor predicted gender ratios

Table 3 examines the concordance between the gender distribution predicted by the NamSor software and the actual participant genders.

**Table 3:** Concordance analysis between actual gender and NamSor-predicted gender

Variables		Actual Gender				*p*	Kappa	Accuracy	Sensitivity	Specificity	Precision
		Female		Male							
		Number	%	Number	%						
Gender (NamSor)	Female	2611	75.1	798	46.4	< 0.001**	0.283	67.9%	75.1%	53.6%	76.0%
	Male	868	24.9	921	53.6						

*p < 0.05, **p < 0.01, 𝜒2: Chi-square test for categorical variables

A statistically significant relationship was found between the gender distribution predicted by the NamSor software and the participants’ actual genders (*p* < 0.05), and a low level of agreement was observed with a Kappa coefficient of 0.283. While 75.1% (*n* = 2611) of individuals whose actual gender was female were correctly predicted as female, 24.9% (*n* = 868) were incorrectly classified as male. Among individuals whose actual gender was male, 53.6% (*n* = 921) were correctly predicted as male, whereas 46.4% (*n* = 798) were incorrectly predicted as female. When reviewing the concordance indicators, the accuracy rate was 67.9%, sensitivity was 75.1%, specificity was 53.6%, and precision was 76.0%. These findings indicate that NamSor is relatively more successful in predicting females, but the lower specificity for male’s results in a higher margin of error.

**Fig. 4:**
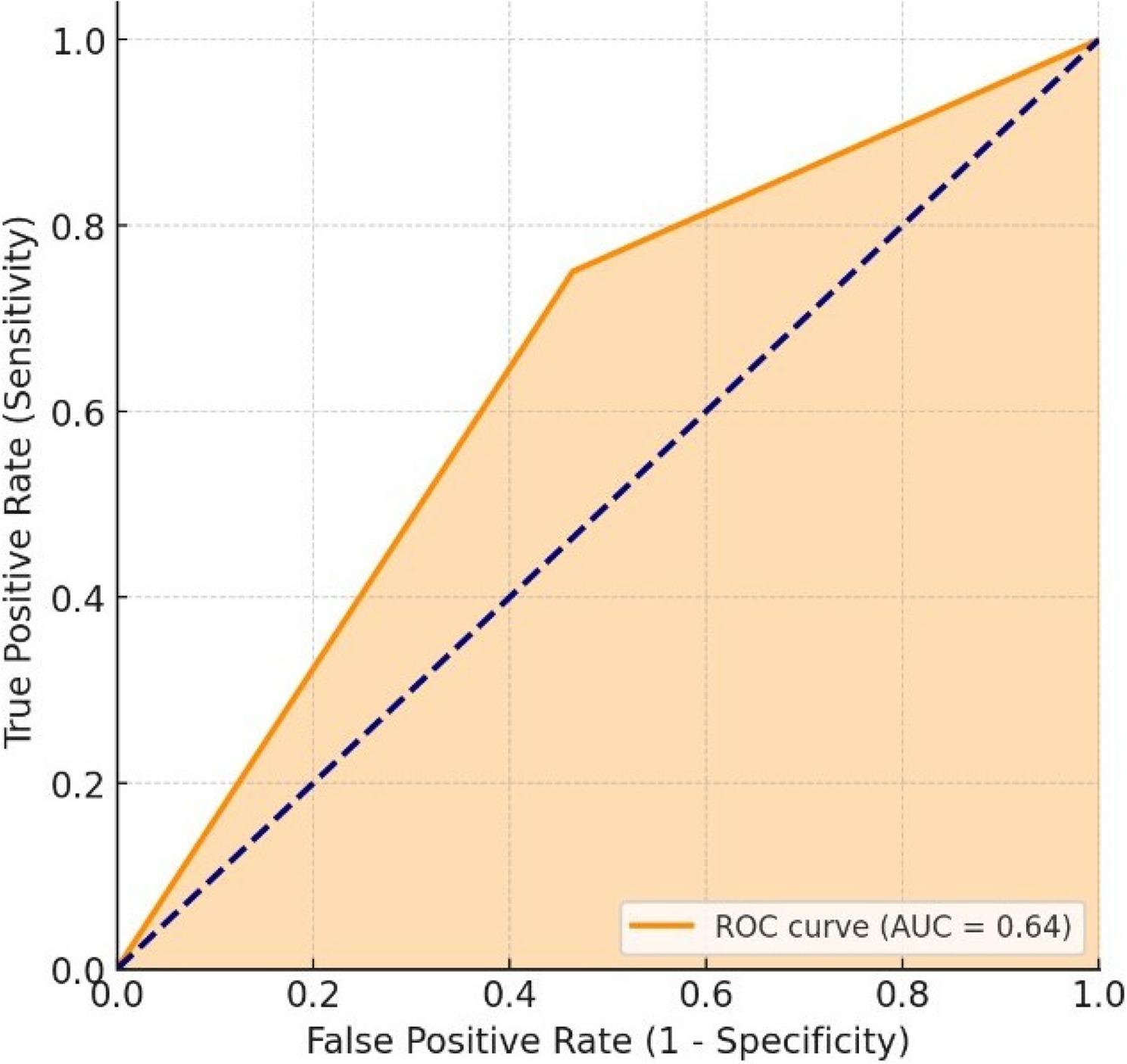
ROC curve showing the agreement between actual gender and NamSor predicted gender ratios (Total sample size: *N* = 5198). The area under the curve (AUC) was 0.64

Figure 4 shows the agreement between the actual gender and the NamSor-predicted gender using the ROC curve. The ROC curve presents the model’s success in correctly classifying females (sensitivity, 75.1%) and its success in correctly identifying males (specificity, 53.6%) on the same graph. The area under the curve (AUC = 0.64) indicates that the model performed better than random classification (AUC = 0.50) but did not have a strong discriminatory power. In other words, while the NamSor software’s success in accurately identifying females is relatively high, the accuracy rate for classifying males is lower. The observed agreement levels suggest that automated gender classification tools should be interpreted cautiously when used in educational workforce analyses. The ROC analysis in this context does not represent diagnostic test performance but rather illustrates the discriminatory capacity of the algorithm in classifying the recorded sex categories within this dataset.

It should be emphasized that all inferential analyses regarding gender distribution across specialties were conducted using manually verified gender data. The NamSor results were presented solely to evaluate the performance limitations of automated gender inference tools in large educational datasets.

## Discussion

This original research comparatively examined the gender distribution of students receiving specialist training in dentistry faculties in Türkiye using actual data and predictions made by the artificial intelligence-based NamSor model. The findings reveal that there are significant differences in specialty preferences between female and male students; these differences appear to reflect not only individual choices but also structural and cultural factors. While inequality reflects measurable differences in representation, inequity refers to the structural conditions sustaining these disparities [1]. Accordingly, the concentration of male students in high-intensity surgical branches may reflect not only a numerical imbalance but also underlying institutional and sociocultural constraints within academic dentistry. The noticeably higher representation of male students in Oral, Dental, and Maxillofacial Surgery—a field with a high surgical workload—demonstrates, consistent with the existing literature, that female underrepresentation in such specialties may be associated with barriers reported in the literature [2, 3, 6, 7]. These differences can be interpreted within a structural and institutional framework, rather than solely as individual preferences. Given the census-based design of this study, statistical significance reflects the observed population-level patterns rather than sampling variability. Demanding working conditions, long hours, and emergency responsibilities in surgical training may disproportionately affect women, particularly regarding caregiving expectations [14, 15]. In contrast, female representation is higher in branches such as Pedodontics and Restorative Dental Treatment, which parallels previous findings that reveal that women tend to prioritize factors such as patient communication, fixed working hours, and work-life balance [4, 5].

Moreover, limited mentorship opportunities and fewer senior female role models may further contribute to this imbalance are other factors reinforcing this gender imbalance [16, 17]. Therefore, specialty choices may be associated not only with individual motivation but also with gender roles in society and institutional inequalities in opportunities [1, 18]. In surgical disciplines such as Oral and Maxillofacial Surgery, where training demands are particularly intensive, these structural dynamics may operate more visibly. The scarcity of senior female role models in such branches may be associated with self-selection patterns and reduce perceived attainability, thereby perpetuating inequitable gender stratification across academic trajectories [16, 17].

NamSor, the artificial intelligence-based gender prediction tool used in the study, was relatively successful in identifying female individuals (75.1% sensitivity, 76.0% precision); however, its accuracy in classifying male individuals was lower. The low agreement level indicated by the Kappa coefficient of 0.283 suggests that such algorithms may be influenced by cultural context and that systematic errors may arise in classifications based on name origin [13]. Nevertheless, the fact that NamSor performed better than random classification (AUC = 0.64) shows that such software may be considered a supplementary exploratory tool in large datasets; however, its performance metrics in this study indicate that verified administrative data remain the gold standard for gender classification. This supports the cautious approach in the literature regarding the use of artificial intelligence applications as supportive tools in the health sciences [19, 20]. In this study, ROC and AUC metrics were used strictly as statistical measures of classification performance and should not be interpreted as indicators of clinical diagnostic validity.

In culturally heterogeneous societies such as Türkiye, where naming conventions may reflect diverse linguistic, ethnic, and historical influences, the risk of misclassification may be increased [18]. Name-based algorithms are typically trained on datasets derived from specific populations, which may limit their generalizability across contexts [19, 20]. Therefore, while NamSor can serve as a supportive analytical instrument in large-scale research, it should not be interpreted as a definitive or individually valid gender classification tool [19]. Importantly, the main conclusions of this study are based on verified administrative records. The NamSor results were presented only as a methodological evaluation of the automated gender inference performance.

Improving gender equity in dental specialty education requires policies that move beyond simply balancing headcounts and instead address structural barriers across the academic pipeline [2, 3]. Global and discipline-specific studies have shown that although women now constitute a substantial share of the dental workforce and student body, they remain underrepresented in senior academic and leadership positions, with persistent gaps linked to limited mentoring opportunities, unequal caregiving responsibilities, and biased promotion processes [21, 22]. Evidence from academic medicine and health professions further indicates that structured mentoring programs, leadership development initiatives, and gender-transformative institutional policies can improve women’s promotion rates, retention, and career satisfaction [23, 24]. Embedding these strategies, together with systematic monitoring of gender indicators, into admissions, postgraduate training, and governance processes in dental schools may be essential to reduce the inequities highlighted in our findings.

The study findings also quantitatively reveal the ongoing gender inequalities in dentistry and suggest that these disparities have a multi-layered structure, extending from the undergraduate level to specialist training and academic advancement processes. Although females are highly represented at the undergraduate level, the literature shows that they are present at lower rates in the upper levels of specialist training and academic positions [2, 3, 9]. This pattern reflects the “leaky pipeline” phenomenon observed in academic health professions, where female representation declines at advanced training and leadership levels, particularly in high-intensity surgical fields [3, 21, 22]. Such attrition may be associated less with individual preference than with structural pressures, including demanding work schedules, caregiving expectations, limited mentorship, and institutional bias [14–17]. The fact that students perceive female instructors as more inclusive, understanding, and supportive serves as an important indicator of the impact of instructor gender on student attitudes and professional orientation [8].

Most superficial equality policies developed in recent years focus solely on numerical gender ratios but fall short of eliminating the underlying cultural and institutional barriers. For true equality, gender-based structural biases in recruitment, promotion, evaluation, and leadership processes must be systematically analyzed and transformed [1, 20]. Inclusive mentoring programs and leadership development support play significant roles in achieving this transformation [16, 17].

Our findings in Türkiye reveal that gender-based inequalities in dental specialty education are not only a local issue but also exhibit similar patterns on a global scale. While this study examined gender distribution across specialties during postgraduate training, it did not directly assess representation in senior academic or leadership positions. Leadership roles constitute a distinct dimension of structural inequity and may reflect the cumulative effects of specialty-level disparities over time [2, 21]. Although the underrepresentation of women in high-intensity surgical branches is prominent, the relatively lower representation of male trainees in certain nonsurgical specialties also warrants consideration, as gendered specialty patterns are shaped by broader sociocultural and institutional factors rather than purely individual preferences [8, 25]. A recent study conducted in Australia reported that the representation of women in dental professional organizations and academic leadership positions was significantly lower than that of men [26]. This confirms the low representation of women in surgical branches in Türkiye.

Beyond international comparisons, national studies have also shed light on gender dynamics within dental postgraduate training in Türkiye. An analysis of theses in Oral and Maxillofacial Surgery programs reported a predominance of male-authored academic work [27]. Likewise, a bibliometric evaluation of endodontics specialization theses demonstrated that although female physicians authored more theses, publication rates were comparable between genders [28]. Together, these findings support the interpretation of gendered patterns in specialty training in the Turkish academic context.

Similar patterns were observed across the medical and surgical specialties. Workforce data indicate that women constitute only approximately one-quarter of residents in fields such as orthopedic surgery and neurosurgery, with even lower representation in senior surgical positions [29]. Reports from Oral and Maxillofacial Surgery also demonstrate persistent gender gaps in training and academic leadership [6]. These parallels suggest that the distribution observed in Türkiye reflects a broader structural trend rather than discipline-specific phenomena.

Similar gender disparities have been documented in Brazil. Martorell et al. reported that women are underrepresented among invited speakers at major dental conferences [30]. Braz et al. demonstrated lower proportions of women in senior authorship positions in oral medicine and pathology [31]. Likewise, de Sousa et al. emphasized that despite recent progress, structural gender inequities persist in Brazilian dental academia [32]. Together, these findings suggest that the patterns observed in Türkiye align with broader international trends rather than representing an isolated phenomenon.

From a global perspective, Tiwari et al., who studied different countries, showed that women in the dental workforce tend to gravitate towards fields that require “less surgical intensity,” such as pedodontics and restorative dentistry. This result largely parallels the distribution of specialties identified in Türkiye [2].

One of the most significant contributions of this study, conducted in Türkiye, is that it provides a comprehensive analysis of both data quality and the predictive power of algorithms by comparing verified gender information obtained from publicly available data with artificial intelligence results. However, certain limitations of this study should not be overlooked. An important limitation of this study is the operationalization of gender within a binary framework (female/male), as determined by the available administrative records. This approach does not capture non-binary or gender-diverse identities and therefore cannot fully represent the complexity of gender in contemporary academic contexts. Future research should aim to incorporate more inclusive gender classifications, where data availability permits. Moreover, the impact of cultural and linguistic differences on the prediction performance of artificial intelligence should be considered as a separate subject of examination.

This study is among the few to address both gender disparities in dental specialty education in Türkiye and the role of AI-based analysis. These findings provide valuable guidance for policymakers, administrators, and academic advisors. Expanding support for women in surgical fields and leadership roles, fostering inclusive mentorship, and implementing equity-driven institutional policies are essential for narrowing these gaps. Moreover, the ethical and cautious use of AI in large-scale data analyses can strengthen research quality and ensure data security.

## Conclusion

This study provides national evidence that gender-based disparities persist in dental specialty education in Türkiye, particularly in surgical fields, such as Oral and Maxillofacial Surgery. Female students were predominantly represented in Pediatric and Restorative Dentistry, reflecting patterns consistent with the structural and cultural factors discussed in the literature. The modest performance of the NamSor algorithm highlights the contextual limitations of AI-based gender identification, thus emphasizing the need for human oversight and cultural calibration. Promoting inclusive mentorship, enhancing women’s visibility in surgical and academic leadership, and integrating gender sensitivity into institutional policies are essential steps toward achieving genuine equity in dental education and professional advancement.

## Data Availability

The datasets used and/or analyzed in the current study were derived from publicly available sources, including official university websites and the Turkish Council of Higher Education. The analyzed datasets are available from the corresponding author upon reasonable requests.
